# Acceptability Evaluation of the Use of Virtual Reality Games in Smoking-Prevention Education for High School Students: Prospective Observational Study

**DOI:** 10.2196/28037

**Published:** 2021-09-28

**Authors:** Jong-Long Guo, Hsiao-Pei Hsu, Tzu-Ming Lai, Mei-Ling Lin, Chih-Ming Chung, Chiu-Mieh Huang

**Affiliations:** 1 Department of Health Promotion and Health Education College of Education National Taiwan Normal University Taipei Taiwan; 2 Department of Nursing College of Nursing National Yang Ming Chiao Tung University Taipei Taiwan; 3 Wanfang High School Taipei Taiwan; 4 Department of Applied Information Technology Hsing Wu University New Taipei Taiwan; 5 Institute of Clinical Nursing College of Nursing National Yang Ming Chiao Tung University Taipei Taiwan

**Keywords:** behavioral intention, ARCS motivation model, persuasiveness, smoking prevention, educational games

## Abstract

**Background:**

Alternative forms of cigarettes, such as electronic cigarettes (e-cigarettes), are becoming increasingly common among adolescents. Many high schools now provide smoking-prevention education in an attempt to minimize the potential negative health effects and illness burdens e-cigarettes may induce in adolescents. However, it is often difficult to motivate young students to engage with traditional education regarding the harmful effects of tobacco; thus, the development of alternative approaches may be required.

**Objective:**

In this study, we aimed to conduct an acceptability evaluation of educational virtual reality games designed to support smoking-prevention measures. We based the acceptability evaluation on the following two experience types: game-playing and content-learning experiences. The paths by which these experience types affect the intention to abstain from smoking were also examined.

**Methods:**

We applied a prospective observational study design. We developed educational games based on three-dimensional virtual reality technology, in which participants operated joysticks to complete challenge tasks. To increase the possibility of the games fostering motivation to abstain from smoking, the ARCS motivational model (comprising attention, relevance, confidence, and satisfaction) was used as a framework during the games’ design. We measured the participants’ game-playing experiences by inquiring about the strength of the ARCS elements; content-learning experiences were measured using overall knowledge improvement and the perceived persuasiveness of the content. A total of 130 students participated in the program. Study hypotheses for this evaluation were derived from a literature review. We used partial least squares structural equation modeling to examine the proposed hypotheses.

**Results:**

Based on the responses of the students to questionnaire items concerning attention, relevance, confidence, and satisfaction in the context of the games, most students agreed or strongly agreed that the educational games were motivational, and that their game-playing experiences were positive. Regarding content-learning experiences, there was a significant improvement in knowledge (*t*_129_=25.67, *P*<.001), and most students perceived themselves as being persuaded to abstain from smoking. Attention, relevance, and satisfaction significantly influenced perceived persuasiveness (*t*=3.19, *P*<.001; *t*=4.28, *P*<.001; and *t*=3.49, *P*<.001, respectively); however, confidence did not (*t*=0.42, *P*=.67). Perceived persuasiveness, relevance, and satisfaction significantly influenced the intention to abstain from smoking (*t*=3.57, *P*<.001). In addition to directly affecting the intention to abstain from smoking, indirect effects were observed from both relevance and satisfaction to intention via perceived persuasiveness (*t*=2.87, *P*=.004 and *t*=2.11, *P=*.04, respectively). However, intention was not significantly influenced by knowledge improvement.

**Conclusions:**

Our findings revealed that the educational games were positively accepted by the participating students. This indicates that the integration of the ARCS framework and persuasive strategies is applicable for smoking-prevention education. We recommend that the games be included as teaching materials for smoking-prevention education.

## Introduction

Smoking causes approximately 7 million deaths worldwide annually and is commonly associated with noncommunicable diseases and disability [1]. Thus, smoking causes great harm to human health, and it can have particularly pronounced adverse effects among adolescents. Adolescence is a developmental stage, and engaging in smoking during this period can impair brain development [2]. In addition, adolescents can be prone to early nicotine dependence symptoms, which can increase the likelihood that they will be addicted to smoking in adulthood [3].

According to the World Health Organization’s Framework Convention on Tobacco Control, it is important to promote and strengthen education and public awareness regarding the harms associated with tobacco [4]. In recent years, the prevalence of electronic cigarette (e-cigarette) use on campuses has rapidly increased; this is occurring despite the fact that there are many unanswered questions regarding the safety of e-cigarettes, such as whether they represent a less-harmful substitute for traditional cigarettes [5]. Further, their effectiveness as a means of facilitating smoking cessation and their overall impact on health, especially among adolescents, are also unclear [5]. Implementing e-cigarette–prevention measures in schools could help to minimize the potential negative health effects and illness burdens e-cigarettes may induce in adolescents. One of the most important school-based e-cigarette–prevention efforts is educating young students regarding the toxic exposure, potential risks, and health effects associated with e-cigarettes [6]. However, it is often difficult to motivate young students to engage with traditional education regarding the harms associated with tobacco; thus, the development of alternative approaches, such as educational games, may be more effective in this regard.

The use of games for health promotion and illness prevention is becoming increasingly common, and previous studies have revealed that gaming-based approaches can promote health behaviors among adolescents [7]. In the context of education, game-based learning (GBL) can be a practical and effective approach. Recently, immersive virtual reality (VR) has been introduced as a medium for learning and teaching, and has had a considerable influence in this regard [8]. Immersive VR allows users to perform actions in a virtual environment and provides opportunities for contextual practice [8]. Notably, conducting GBL through immersive VR enables situational learning, with learners using devices, such as helmets and joysticks, to observe and interact with virtual educational scenes [9,10]. Its characteristic of providing an interactive learning environment means this approach could increase learners’ engagement with the content. This is notable because researchers have suggested that a lack of engagement may undermine learning effectiveness [11]. Further, studies have found that it is important to present engaging content as early as possible, and educational games quickly engage young students [12]. Enhancing engagement may increase the likelihood of favorable learning outcomes in which learners develop sufficient motivation to perform the target behavior [13]. This indicates that educational games may be an effective alternative option or supplementary material for smoking-prevention courses in schools.

In Taiwan, health education is a compulsory subject for high school students, and smoking prevention is one of the mandated learning contents stipulated in the national basic K-12 education curriculum [14]. The present researchers developed VR games to function as supplementary material for smoking-prevention education. School teachers may integrate these games into health education in a manner that suits their students and circumstances. Despite the potential benefits of educational games, the acceptability of educational VR games remains an underresearched area. Consequently, this study concerns an acceptability evaluation of educational VR games for smoking prevention, rather than an evaluation of the effectiveness of including VR games as part of a smoking-prevention program. Enhancing the understanding of the acceptability of VR games in this regard could help improve the design of such games and learners’ engagement, thereby increasing the effectiveness of the education.

In this study, we based the acceptability evaluation on the following two experience types: game-playing and content-learning experiences. We derived measures of game-playing experiences from Keller’s ARCS (attention, relevance, confidence, and satisfaction) motivation model [15]. To achieve effective learning, motivating learners is an important first step; however, scholars have suggested that motivation is unpredictable and changeable [16]. Thus, a systematic method of identifying motivation is essential for enhancing learning effectiveness. The ARCS motivation model provides a framework for understanding motivation. According to the ARCS model, learners who experience attention, relevance, confidence, and satisfaction through a learning process are likely to be motivated in regard to the educational content in question [17,18]. Therefore, in this study, we applied the ARCS model to assess students’ game-playing experiences. We expected students to be motivated by playing the games, and sought to determine their game-playing experiences through ARCS assessment.

Content-learning experiences are defined as the knowledge obtained from educational material and the perceived persuasiveness of educational material. Behavioral knowledge is essential for behavior changes; without proper knowledge and guidance, it can be difficult to adopt a specific behavior [19]. However, knowledge alone is unlikely to generate behavior change [20], and a more integrated approach is needed. Thus, learners’ perceptions of the persuasiveness of content are also important. The perceived persuasiveness of a system is an estimation of its ability to motivate behavior change [21].

Persuasive system design includes behavior change strategies that support learners in attaining the target behavior. The following four features have been suggested for persuasive system design: task support (ie, self-monitoring), dialogue support (ie, positive reinforcement), credibility (ie, conveying trustworthiness), and social support (ie, provision of social information) [22]. Scholars have suggested several behavioral intervention strategies, such as providing information and instructions to increase learners’ understanding and abilities to achieve a goal, anticipation of barriers, monitoring and feedback, social comparison, and rewards [23].

The educational VR games were designed to convey messages relating to smoking prevention. In this context, effective content learning should induce, among learners, enhanced knowledge of smoking prevention and a higher likelihood (through perceived persuasion) of adopting smoking-prevention behaviors.

Educational games for health are designed to motivate individuals to improve their health through implementing behavior changes. In this study, the target change was developing an intention to abstain from smoking. Behavioral intentions have a strong causal impact on behavior [24], and we expected that strengthening students’ intentions to abstain from smoking would decrease their risk of adopting smoking behaviors in the future. Students’ engagement with the games should produce both game-playing and content-learning experiences, and these two types of experiences should influence students’ intentions to abstain from smoking. Based on this, we proposed the following hypotheses: hypothesis 1 (H1), attention influences behavioral intention; hypothesis 2 (H2), relevance influences behavioral intention; hypothesis 3 (H3), confidence influences behavioral intention; hypothesis 4 (H4), satisfaction influences behavioral intention; hypothesis 5 (H5), knowledge influences behavioral intention; and hypothesis 6 (H6), perceived persuasiveness influences behavioral intention.

**Figure 1:**
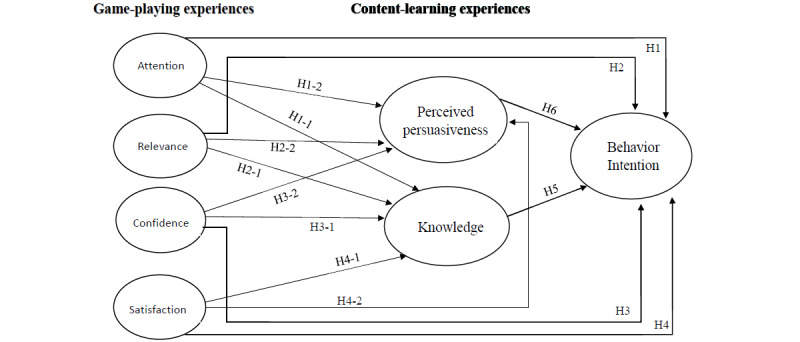
The hypotheses of the proposed model.

Our educational VR games were designed to not only be fun, but also improve health. They represent digital persuasion agents that affect learners’ engagement and, hopefully, motivate learners to change their behaviors. Notably, effective learning occurs through successful persuasion, which is a product of positive changes in cognition and attitude [25]. Previous studies have suggested that engagement with digital technology (in this study, “game-playing experiences”) impacts the quality of users’ experiences with the technology [26]. Effective game-playing experiences are necessary to enhance learners’ engagement with the learning process. We propose that game-playing experiences cause learners to engage with learning content, which consequently creates a content-learning experience for the learners. Thus, we developed the following hypotheses regarding the influence of game-playing experiences on content-learning experiences: hypothesis 1-1 (H1-1), attention influences knowledge; hypothesis 1-2 (H1-2), attention influences perceived persuasiveness; hypothesis 2-1 (H2-1), relevance influences knowledge; hypothesis 2-2 (H2-2), relevance influences perceived persuasiveness; hypothesis 3-1 (H3-1), confidence influences knowledge; hypothesis 3-2 (H3-2), confidence influences perceived persuasiveness; hypothesis 4-1 (H4-1), satisfaction influences knowledge; and hypothesis 4-2 (H4-2), satisfaction influences perceived persuasiveness. All the hypotheses of the proposed model are summarized in Figure 1.

## Methods

### Participants and Recruitment

This study featured a prospective observational study design. The participating students (1) were high school students, (2) did not have any major chronic diseases (eg, asthma, heart disease, and diabetes), (3) were not pregnant, (4) were willing to comply with verbal instructions, and (5) were able to provide written informed consent.

We conducted this study among students from a high school in Taipei City, Taiwan. The research team made an appointment with the school personnel who were responsible for implementing smoking-prevention measures on the school campus. During the meetings, we distributed study information and discussed with the school personnel potential barriers in regard to the implementation of the materials. Once a consensus was reached in regard to the manner by which the materials would be implemented, the school personnel announced the research project to students and distributed an information sheet. Students who were willing to participate were informed by the school personnel that the research sought to measure students’ responses toward educational games. Participating students received a small gift as compensation for their contribution to smoking-prevention research.

### Design of the Educational VR Games

We designed the educational VR games to function as supplementary material for smoking-prevention education. The theoretical concepts underlying the educational games’ designs are depicted in Multimedia Appendix 1. We based the games’ designs on the ARCS model of motivation (Multimedia Appendix 1); this model was developed by Dr John Keller [15], who designed the model to identify the important contributors to students’ motivation. In addition, we presented the motivating concepts in the educational games in a manner that allowed them to correspond with specific persuasive strategies.

The educational VR games included a whack-a-mole game, a wire loop game, a square baseball game, and a Taiko drum game. Participating students wore a head-mounted display (HMD), which enabled them to have immersive experiences with 3D images. To complete the challenge tasks, the participants interacted with the virtual environments through the use of joysticks; they also received in-game guidance from an avatar.

### Measurements

We used questionnaires to collect research data; these data included the students’ background information, their perceived acceptability of the VR games, and their intention to abstain from smoking. The questionnaires were reviewed by four professionals, including two senior health-education teachers, one professor of health education, and one professor of nursing. We used the content validity index (CVI) to evaluate whether the items were appropriate for measuring the study concepts; only items with CVI values >0.80 were retained [27]. One item pertaining to confidence, “I found the games difficult,” was deleted because of low factor loading (<0.6). This question was the only reverse-scored item in the questionnaire, which may have been the reason for the low factor loading.

#### Background Information and Intention to Abstain From Smoking

We collected background information for each participant, including their sex, age, whether they lived with both parents, their personal smoking experience, and their parents’ and peers’ smoking status. We performed the acceptability evaluation by measuring their game-playing and content-learning experiences. We expected that these experiences would strengthen the students’ intention to abstain from smoking. Thus, the primary outcome of this study was students’ intention to abstain from smoking, which was measured using two items; these were scored using a Likert-type scale ranging from 1 (“strongly disagree”) to 5 (“strongly agree”), with higher scores indicating higher levels of intention. The study variables, corresponding items, and convergent validity values are presented in Table 1.

**Table 1:** Study variables, corresponding items, and convergent validity.

Experiences and variables				Corresponding items		Factor loading		CR^a^		AVE^b^
**Game-playing experiences^c^**										
	**Attention**							0.93		0.77
		Atten 1	The games’ virtual reality scenes were eye-catching.		0.86					
		Atten 2	The educational games provoked my curiosity.		0.87					
		Atten 3	The interactional elements of the games held my attention.		0.90					
		Atten 4	The educational games were fun to play.		0.88					
	**Relevance**							0.87		0.62
		Relev 1	The content presented in the games was relevant to my needs.		0.84					
		Relev 2	The content presented in the games was relevant to my interests.		0.66					
		Relev 3	The content presented in the games was useful to me.		0.76					
		Relev 4	I was familiar with the content presented in the games.		0.87					
	**Confidence**							0.80		0.57
		Confi 1	I was confident I could complete the tasks in the games.		0.71					
		Confi 2	My successes in the games were a result of my own efforts.		0.70					
		Confi 3	I could effectively operate the joysticks to interact with the virtual reality scenes.		0.86					
	**Satisfaction**							0.86		0.61
		Satis 1	I could apply the content conveyed in the games in real life.		0.80					
		Satis 2	I benefitted by playing the games.		0.76					
		Satis 3	I felt a sense of accomplishment after finishing the tasks in the games.		0.82					
		Satis 4	I was satisfied with my performance in the games.		0.73					
**Content-learning experiences^d^**										
	Knowledge improvement			Fifteen true/false type questions related to smoking prevention were used to measure knowledge. Improvement was defined as postscore minus prescore.		1.00		1.00		1.00
	**Perceived persuasiveness**							0.91		0.70
		Persu 1	The games enhanced my awareness of smoking prevention.		0.82					
		Persu 2	The games enhanced my concern regarding smoking prevention.		0.81					
		Persu 3	The games enhanced my understanding of smoking-prevention methods.		0.86					
		Persu 4	The games enhanced my self-efficacy in regard to practicing smoking-prevention skills.		0.86					
	**Intention to abstain from smoking**							0.95		0.91
		Inten 1	The content of the games has made me consider abstaining from smoking.		0.95					
		Inten 2	In the future, I will abstain from smoking by using the content of the games as a reference.		0.96					

^a^CR: composite reliability. The CR criterion was >0.7 [26].

^b^AVE: average variance extracted. The AVE criterion was >0.5 [26].

^c^Motivation concepts were used to measure the game-playing experiences.

^d^Knowledge improvement and perceived persuasiveness were used to measure the content-learning experiences.

#### Acceptability Evaluation: Game-Playing Experiences

We designed the educational games to improve students’ motivation. Therefore, we used motivation concepts (ARCS) to measure the game-playing experiences. Specifically, this study intended to examine the educational VR games’ effectiveness as a teaching medium in terms of the games’ ability to focus learners’ attention, stimulate learning confidence, and effectively improve learning satisfaction. We based the measurement items on existing conceptualizations of the ARCS motivation model [13]. Attention was defined as follows: “Students’ attention is directed to the stimuli, and their interest is maintained over time.” Relevance was defined as follows: “Students perceive the contents as being related to their real-life experiences.” Confidence was defined as follows: “Confident students attribute their successes in the games to their own ability and efforts.” Satisfaction was defined as follows: “Students perceived the education as inducing internal feelings of satisfaction.” We measured each aspect using four items, all of which were scored using a Likert-type scale ranging from 1 (“strongly disagree”) to 5 (“strongly agree”), with higher scores indicating higher levels of motivation. Details of the ARCS measures are shown in Table 1.

#### Acceptability Evaluation: Content-Learning Experiences

In addition to motivating students through game-playing experiences, we expected that the educational VR games would, through content-learning experiences, influence the students’ perspectives of smoking. The strength of the content-learning experiences was measured by examining knowledge improvement and perceived persuasiveness.

To measure knowledge improvement, we presented 15 true/false-type questions related to smoking prevention to the participants. Through administering pretests and posttests, we determined changes in the participants’ scores between the pre-experiment and postexperiment periods.

Perceived persuasiveness was defined as students’ awareness of and concern regarding smoking prevention, as well as their understanding of and self-efficacy regarding practicing preventive skills. We used four items to measure perceived persuasiveness (Table 1), with higher scores indicating higher levels of perceived persuasiveness.

### Data Collection Procedure

This study received approval from the Research Ethics Review Committee of En Chu Kong Hospital (ECKIRB1090401). All of the participating students expressed their approval of the study and provided written informed consent. First, research staff gave the participants brief instructions regarding the educational VR games. Then, before playing, students completed a questionnaire that measured their knowledge of smoking prevention. The research staff then gave the students guidance regarding how to use the HMDs and joysticks, which enabled them to have immersive interactive experiences with 3D images. Students who agreed to participate in the research received the educational VR games to be played outside of class. The students were allowed to play the games freely; there was no fixed order regarding the games played. It takes about 35 to 45 minutes to complete the educational tasks. Health education is a compulsory course in Taiwan. Substance use prevention, including smoking prevention, is one of the required topics of the course. However, these students did not receive a similar course during the research. They learned about substance use prevention during a different semester. Research staff were present while the students completed the games, but did not provide any oral instructions. Once the students completed the educational games, the research staff provided them with a structured questionnaire that evaluated the acceptability of the games and their intention to abstain from smoking.

### Data Analysis

We performed statistical analysis using SPSS 23.0 (IBM Corp). The participants’ characteristics were described using numbers and percentages. Means and standard deviations were used to represent the variables associated with the games’ acceptability. To examine the proposed hypotheses, we conducted partial least squares structural equation modeling (PLS-SEM) using the SmartPLS v3.0 program (SmartPLS GmbH); this approach was chosen because PLS-SEM is suitable for creating exploratory models and for research involving small sample sizes [28]. Using parameters with a significance level of .05, a statistical power of 80%, and an R^2^ value of at least 0.25, the suggested sample size was 75 for the maximum number of six arrows pointing to a latent variable in the model [29]. Thus, a sample size of 130 in this study was sufficient.

PLS‑SEM does not presume that the data are normally distributed. The process of bootstrapping involves repeated random sampling with replacement from the original sample to create a bootstrap sample. Using bootstrapping assumes that the sample distribution is a reasonable representation of the intended population distribution. The minimum number of bootstrap samples is 5000 [28]. The bootstrap sample enables the estimated path coefficients in PLS‑SEM to be tested for their significance. Significant paths showing the hypothesized direction empirically supported the proposed relationship.

We conducted PLS-SEM in the following two steps: (1) assessment of the reliability and validity of the measurement model, which was used to test the relationships between each latent variable and its indicators; and (2) assessment of the structural model, through which estimates were provided for the path coefficients, which represented the hypothesized relationships among the latent variables in the proposed model [30,31].

#### Measurement Model

We examined the convergent and discriminant validity in order to verify that the latent variables (the game-playing and content-learning experiences and intention to abstain from smoking) were valid and reliable.

Convergent validity can be determined by considering composite reliability (CR) and average variance extracted (AVE) values. To meet the convergent validity and reliability requirements for the model, CR should be greater than 0.7 [32] and AVE should be greater than 0.5 [28]. We used CR to evaluate the construct measures’ internal consistency reliability. Using CR to measure internal consistency reliability meant that the PLS-SEM could accommodate different indicator reliabilities and that the underestimation associated with Cronbach α was avoided [28]. Meanwhile, AVE indicates the latent variables’ accounted variance from the study measures, and higher AVE values indicate higher accounted variance.

To establish discriminant validity, we examined the Fornell-Larcker criterion [28] and the heterotrait-monotrait (HTMT) [32]. To satisfy the Fornell-Larcker criterion, the square root of the AVE for each variable should exceed the correlation of the latent variables [28]. Meanwhile, for the HTMT, values of <1.00 indicate discriminant validity [32].

#### Structural Model

PLS-SEM indicates explained variances for the latent variables through R^2^ (coefficient of determination), which describes levels of predictive accuracy. Estimates (β coefficient) were provided for the path coefficients to indicate the strength of the relationship between the latent variables [31].

To test the significance of the relationship between the latent variables, we estimated *t* values and reported their corresponding *P* values [31]. We used the standardized root mean square residual (SRMR) to test the model’s fit (<0.08 indicates a good fit [32]).

## Results

### Participants’ Characteristics

The participants (N=130) comprised 57 (43.8%) male and 73 (56.2%) female students. The mean age of the sample was 16.64 years, and most participants (113/130, 86.9%) were under 17 years of age. Over half (102/130, 78.5%) lived with both parents. Approximately 20.8% (27/130) of the students had experience of smoking; this included any tobacco product or e-cigarette. Over 30% had family members (45/130, 34.6%) and close friends (41/130, 31.5%) who were smokers (Table 2).

**Table 2:** Participants’ characteristics (N=130).

Characteristic		Value
**Sex, n (%)**		
	Male	57 (43.8)
	Female	73 (56.2)
Age (years), mean (SD)		16.64 (0.73)
**Age group (years), n (%)**		
	≤17	113 (86.9)
	≥18	17 (13.1)
**Household status, n (%)**		
	Live with both parents	102 (78.5)
	Other	28 (21.5)
**Have you ever smoked? (any tobacco product or e-cigarette), n (%)**		
	Yes	27 (20.8)
	No	103 (79.2)
**Do any of your family members smoke?, n (%)**		
	Yes	45 (34.6)
	No	85 (65.4)
**Do any of your close friends smoke?, n (%)**		
	Yes	41 (31.5)
	No	89 (68.5)

### Game-Playing and Content-Learning Experiences

We applied the following two measures to examine the acceptability of the games: game-playing and content-learning experiences. Table 3 shows the distribution of ARCS elements (representing game-playing experiences), knowledge improvement and perceived persuasiveness (representing content-learning experiences), and intention to abstain from smoking (outcome). The item average scores for these variables (defined as the total score divided by the number of items) revealed that the participants provided positive responses. The ARCS-related responses indicated that most students agreed or strongly agreed that the educational games were motivational. The item average scores for attention, relevance, confidence, and satisfaction were all approximately 4 (measured using a Likert-type scale ranging from 1 to 5; scores for the four variables ranged from 3.95 to 4.19). There was a significant improvement in knowledge, with a mean difference of 2.97 between pretest and posttest (*t*_129_=25.67, *P*<.001). Most students indicated that they were persuaded, with an item average score of 4.28 points (determined using a Likert-type scale ranging from 1 to 5).

**Table 3:** Descriptive results for the acceptability evaluation and intention to abstain from smoking.

Game-playing experiences		Item average scores	Sum of mean scores	SD
**ARCS**				
	Attention (four items)	4.19	16.76	2.75
	Relevance (four items)	3.95	15.78	2.70
	Confidence (three items)	4.03	12.08	1.84
	Satisfaction (four items)	4.10	16.38	2.52
**Content-learning experiences**				
	Knowledge improvement (postscore−prescore)	N/A^a^	2.97^b^	1.39
	Perceived persuasiveness (four items)	4.25	17.00	2.48
**Outcome measure**				
	Behavioral intention (two items)	4.18	8.36	1.85

^a^N/A: not applicable.

^b^Mean score for the difference between pretest and posttest.

### Measurement Model Assessment

We established the convergent validity of the latent variables through consideration of CR and AVE. All variables in the model displayed acceptable internal consistency, as evidenced by the fact that their CR scores were greater than 0.7 (ranging from 0.80 to 0.96), and all AVE values were higher than 0.5 (ranging from 0.57 to 0.92). Thus, it was concluded that the model had satisfactory convergent validity (Table 1). The values of these indices satisfied the recommended criteria and suggested that the proposed measurement model was reliable and valid.

As shown in Tables 4 and 5, we established the discriminative validity using the Fornell-Larcker criterion and HTMT. The Fornell-Larcker criterion was satisfied, as the square root of AVE for each variable exceeded the correlation of the latent variables. Almost all of the HTMT values were <1.00 [32], with one of the HTMT values being 1.06, indicating that this criterion was acceptable.

**Table 4:** Results for the Fornell-Larcker criterion.

Variable		Attention	Relevance	Confidence	Satisfaction	Knowledge	Perceived persuasiveness	Behavior intention
**Attention**								
	Latent variable correlation	0.88^a,b^	0.61	0.59	0.65	0.05	0.66	0.42
**Relevance**								
	Latent variable correlation	0.61	0.79	0.60	0.62	−0.03	0.70	0.49
**Confidence**								
	Latent variable correlation	0.59	0.60	0.76	0.76	0.05	0.62	0.41
**Satisfaction**								
	Latent variable correlation	0.65	0.62	0.76	0.78	0.01	0.70	0.51
**Knowledge**								
	Latent variable correlation	0.05	−0.03	0.05	0.01	1.00	−0.01	0.03
**Perceived persuasiveness**								
	Latent variable correlation	0.66	0.70	0.62	0.70	−0.01	0.84	0.60
**Behavior intention**								
	Latent variable correlation	0.42	0.49	0.41	0.51	0.03	0.60	0.95

^a^In SmartPLS output, average variance extracted is calculated and written on the diagonal of the table.

^b^There are no *P* values to provide.

**Table 5:** Results for the heterotrait-monotrait ratio of correlations.

Variable		Attention	Relevance	Confidence	Satisfaction	Knowledge	Perceived persuasiveness	Behavior intention
**Attention**								
	Value	N/A^a,b,c^	0.70	0.77	0.76	0.06	0.75	0.46
**Relevance**								
	Value	0.70	N/A	0.82	0.74	0.07	0.83	0.56
**Confidence**								
	Value	0.77	0.82	N/A	1.06	0.06	0.83	0.52
**Satisfaction**								
	Value	0.76	0.74	1.06	N/A	0.03	0.84	0.59
**Knowledge**								
	Value	0.06	0.07	0.06	0.03	N/A	0.04	0.03
**Perceived persuasiveness**								
	Value	0.75	0.83	0.83	0.84	0.04	N/A	0.67
**Behavior intention**								
	Value	0.46	0.56	0.52	0.59	0.03	0.67	N/A

^a^N/A: not applicable.

^b^The heterotrait-monotrait ratio is the geometric mean of the heterotrait-heteromethod correlations (ie, the correlations of indicators across constructs) divided by the average of the monotrait-heteromethod correlations (ie, the correlations of indicators within the same construct).

^c^There are no *P* values to provide.

### Structural Model Assessment

**Figure 2:**
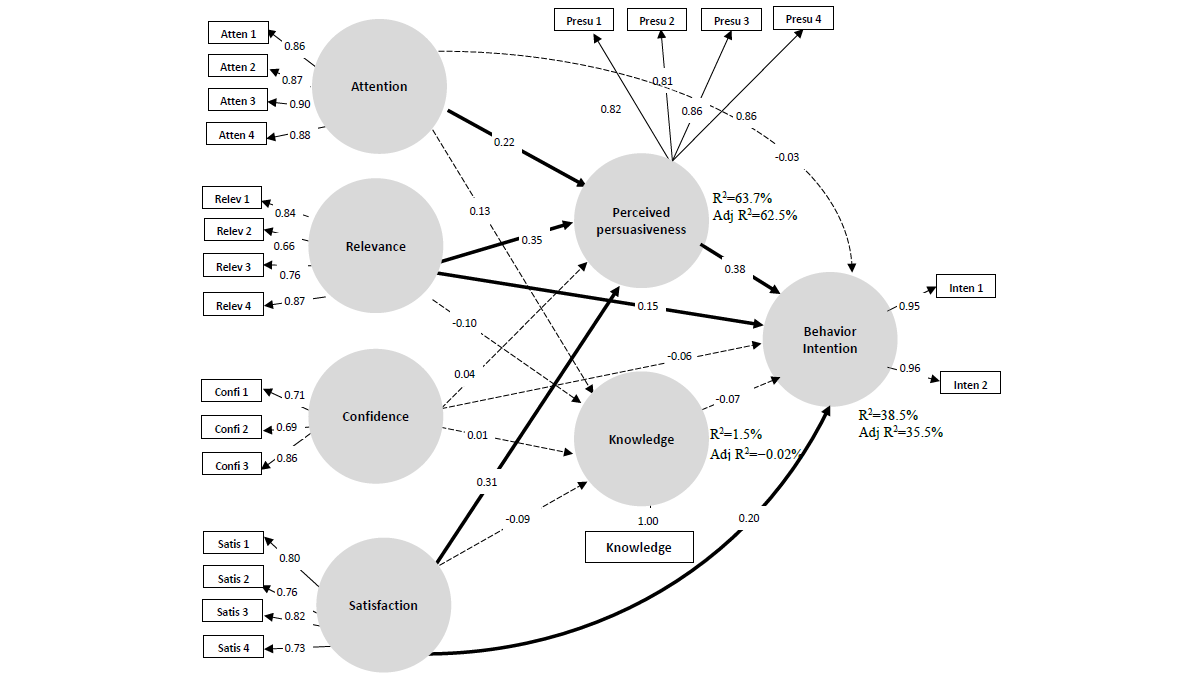
Model for acceptability evaluation of educational virtual reality games.

The model exhibited adequate correspondence with the data (SRMR=0.075). To assess the structural model, we used the *R*^2^ values and the path coefficients as the essential measures. As shown in Figure 2, the four latent variables of ARCS (attention, relevance, confidence, and satisfaction) accounted for 63.7% of the variance in perceived persuasiveness. However, these four latent variables accounted for only 1.1% of the variance in knowledge improvement. Overall, the four latent variables of ARCS, perceived persuasiveness, and knowledge improvement accounted for 38.2% of the variance in the intention to abstain from smoking.

Regarding the path analysis, Table 6 lists the path coefficients and *P* values for each hypothesis. The results of the hypothesis test showed that six of the 14 hypotheses were supported. These six supported hypotheses postulated that the three latent variables of attention, relevance, and satisfaction significantly influenced perceived persuasiveness (H1-2: path coefficient=0.23, *t*=3.19, *P*<.001; H2-2: path coefficient=0.35, *t*=4.28, *P*<.001; H4-2: path coefficient=0.31, *t*=3.49, *P*<.001). Only confidence did not influence perceived persuasiveness (H3-2: path coefficient=0.04, *t*=0.42, *P*=.67). In addition, relevance, satisfaction, and perceived persuasiveness significantly influenced the intention to abstain from smoking (H2: path coefficient=0.26, *t*=2.39, *P*=.02; H4: path coefficient=0.34, *t*=3.07, *P*=.002; H6: path coefficient=0.45, *t*=3.57, *P*<.001). In contrast, intention was not significantly influenced by knowledge improvement (H5: path coefficient=−0.06, *t*=1.31, *P*=.19).

In addition to the direct effect on the intention to abstain from smoking, an indirect effect was observed from relevance to intention via perceived persuasiveness (indirect effect, *t*=2.87, *P*=.004). Further, another indirect effect was observed from satisfaction to intention via perceived persuasiveness (indirect effect, *t*=2.11, *P=*.04). Finally, although not directly affecting the intention to abstain from smoking, attention showed an indirect effect on intention via perceived persuasiveness (indirect effect, *t*=2.16, *P=*.03).

**Table 6:** Results of hypothesis tests.

Hypothesis	Hypothesized paths	Original coefficient	Bootstrapped coefficient	*t*	*P* value	Path support
H1	Attention → behavioral intention	0.06	0.06	0.51	.61	No
H2^a^	Relevance → behavioral intention	0.27	0.26	2.39	.02	Yes
H3	Confidence → behavioral intention	−0.05	−0.04	0.42	.68	No
H4^a^	Satisfaction → behavioral intention	0.34	0.34	3.07	.002	Yes
H5	Knowledge → behavioral intention	−0.07	−0.06	1.31	.19	No
H6^a^	Perceived persuasiveness → behavioral intention	0.44	0.45	3.57	<.001	Yes
H1-1	Attention → knowledge	0.13	0.12	0.78	.44	No
H1-2^a^	Attention → perceived persuasiveness	0.22	0.23	3.19	<.001	Yes
H2-1	Relevance → knowledge	−0.11	−0.10	0.92	.36	No
H2-2^a^	Relevance → perceived persuasiveness	0.35	0.35	4.28	<.001	Yes
H3-1	Confidence → knowledge	0.01	0.01	0.06	.95	No
H3-2	Confidence → perceived persuasiveness	0.04	0.04	0.42	.67	No
H4-1	Satisfaction → knowledge	−0.09	−0.09	0.50	.62	No
H4-2^a^	Satisfaction → perceived persuasiveness	0.31	0.31	3.49	<.001	Yes

^a^Paths with significant coefficients.

## Discussion

### Acceptability Evaluation

In this study, we aimed to evaluate the acceptability of smoking-prevention–focused educational VR games among high school students. We measured acceptability based on the following two aspects: game-playing and content-learning experiences. As motivation is essential for effective learning [33], we adopted the ARCS motivation model as a framework for examining the students’ game-playing experiences. Our findings indicated that the educational games invoked a noticeable degree of learning motivation. Based on the responses of the students to the ARCS-related items, most students agreed or strongly agreed that the educational games were motivational. Many researchers have applied the ARCS model as a framework for instructional design, and the efficacy of the ARCS framework for enhancing students’ motivation has been validated [34,35]. Further, a previous study supported the effectiveness of applying the ARCS framework in the context of learning activities by successfully using it in a randomized controlled trial [36]; the applicability of the ARCS framework for new digital technology, such as mobile augmented reality, has also been proven [37]. Our findings are consistent with those of previous studies and revealed that the educational games could function as supplementary material that engages students and motivates them to learn. The content-learning experiences included in the games comprised knowledge improvement and perceived persuasiveness. We found that the students’ knowledge had significantly improved as a result of playing the VR games. Further, similar to the item average scores for ARCS, most students agreed or strongly agreed that the games had persuaded them to improve their attitudes regarding smoking prevention.

We used persuasive strategies in the design of the human-computer interactions in the games. Several persuasive strategies have been suggested across previous studies [38,39]; however, our findings indicate that strategies concerning competition, self-awareness, feedback, social comparison, and reinforcement are effective in the context of smoking prevention among high school students. Our findings also revealed that the games’ content-learning experiences were influenced by the game-playing experiences. Researchers have emphasized that, to achieve favorable learning outcomes, effective engagement should be pursued over mere passive engagement [40]; theoretically, the students who played our games were motivated to learn through ARCS experiences [41]. The ARCS framework comprises not only instructional approaches to motivate students, but also preliminary elements of effective content learning. We expected that motivated students would show better results in terms of content learning (ie, knowledge improvement and perceived persuasiveness). The study findings consequently showed that attention, relevance, and satisfaction had notable effects on content learning, but confidence did not. Specifically, for the educational games, students who reported high levels of attention, relevance, and satisfaction perceived themselves as being persuaded. Persuasion is a process that concerns changing or reinforcing attitudes or behaviors [24]. Our educational games were designed to discourage students from smoking by reinforcing nonsmoking attitudes and behaviors. In other words, we tried to influence students’ behaviors through messages conveyed in the games. After playing the games, the students reported increased awareness of and concern regarding smoking prevention. In addition, their understanding and self-efficacy in relation to practicing preventive skills were enhanced.

Our findings revealed that only some of the four aspects of motivation (ARCS) contribute to perceived persuasiveness, namely, attention, relevance, and satisfaction. Similar to our findings, attention and relevance have previously been found to be associated with overall persuasiveness [21]. Thus, the present and existing findings imply that, in order to effectively persuade learners, the inclusion of attention and relevance in game design should be prioritized. Further, we found that students who were satisfied with the educational games tended to be persuaded, indicating that satisfaction should also be included in future game design. In contrast, confidence was not an influencing factor; a possible reason for this is that students felt the game tasks were not very challenging and were easy to complete. Some students suggested speeding up the games or increasing the difficulty or number of levels.

The ARCS experiences did not have significant effects on knowledge improvement. In other words, students’ levels of knowledge were not influenced by the game-playing experiences. A possible reason the games nevertheless induced significant knowledge improvement is that the students received written materials while they were waiting in line to play the educational games. Our findings indicate the necessity of integrating other learning strategies into educational games in order to obtain knowledge improvement. As the games function as supplementary material for smoking-prevention education, game playing alone may not be sufficient to influence knowledge. Further, there are conflicting findings in the literature regarding the association among knowledge, positive attitudes, and intent to take action [42,43]. The structural model revealed that knowledge improvement does not influence the intention to abstain from smoking. Consequently, in addition to knowledge of the hazards of smoking, we recommend, for future game development, the inclusion of an emphasis on changing attitudes regarding smoking [44]. However, the gap between knowledge and behavior intention requires further study. Knowledge improvement was viewed as a mediator in the proposed model. Considering knowledge improvement as parallel to behavioral intention rather than a mediator is an idea for further studies.

Once a student’s learning motivation is aroused, how does the level of motivation affect subsequent attitude changes or the achievement of learning objects? We examined this mechanism by analyzing the path from ARCS elements to intention to abstain from smoking. The results revealed that the four aspects of motivation (ARCS) had different influencing paths to intention. In addition to directly influencing students’ intention to abstain from smoking, relevance and satisfaction had indirect effects on intention via perceived persuasiveness. Although the level of attention did not directly influence intention, it had an indirect effect on intention via perceived persuasiveness. These findings indicate that perceived persuasiveness has mediating effects on motivation’s influence on intention. To discourage students from smoking, along with fostering their motivation to learn, perceived persuasiveness should be taken into consideration.

### Implementation

Future efforts to design educational games should incorporate theoretical frameworks that can advance the understanding of the influencing mechanisms for motivation. In this research, we proposed the integration of the ARCS framework and persuasive strategies, and the PLS-SEM results consequently revealed that game-playing experiences that are based on the ARCS framework have direct effects on persuasiveness. Future educational game–focused research could adopt this integrative approach. Second, teaching staff who provide smoking-prevention education in schools could seek to make these VR games as accessible for students as possible, such as through holding activities in fair stalls on school days or during orientation for first-year students. Interesting games can be used to increase students’ awareness of smoke-free campus policies and strengthen students’ commitment to abstain from smoking. Finally, knowledge alone is unlikely to generate behavioral intention; thus, designers of smoking-prevention activities should seek to expand their focus beyond the improvement of knowledge. In addition, a possible problem with true/false questions is that respondents can easily guess the correct answer [45]. Multiple choice questions should be adopted in the future to measure knowledge.

### Limitations

There are several limitations in this study. First, our investigation of the influencing paths for behavior intention was performed in the context of smoking prevention (an unhealthy behavior). While we were certain of the applicability of the ARCS framework and persuasive strategies to educational games that focused on such behaviors, the findings may not be fully generalizable to contexts concerning healthy behaviors. Thus, they should be applied with caution. Second, we could not determine the separate effects of each educational game on behavioral intention, as the participants’ game-playing experiences were obtained as a whole, rather than in terms of each type of game. Third, we could not validate the intervention effects of the educational games, as no comparison group was involved. Finally, one of the HTMT values was larger than 1.00. This implies that two latent constructs may be conceptually similar. Despite these limitations, this study, which was intended as an acceptability evaluation, elaborated on the mechanisms of game-playing and content-learning experiences in the context of behavior intention. We recommend that this experiment design be adopted in future endeavors.

### Conclusion

In this study, we evaluated the acceptability of educational VR games for use in smoking-prevention education for high school students. Our findings revealed that the educational games were positively accepted by the participating students, and that the integration of the ARCS framework and persuasive strategies is an applicable approach for smoking-prevention education. The ARCS framework was significantly associated with perceived persuasiveness, and perceived persuasiveness has direct effects on the intention to abstain from smoking. We recommend that the games be included as teaching material in smoking-prevention education.

## Appendix

Multimedia Appendix 1 Theoretical concepts used in the design of the educational games.

## Abbreviations

ARCS: attention, relevance, confidence, and satisfaction
AVE: average variance extracted
CR: composite reliability
CVI: content validity index
e-cigarette: electronic cigarette
GBL: game-based learning
HMD: head-mounted display
HTMT: heterotrait-monotrait
PLS-SEM: partial least squares structural equation modeling
SRMR: standardized root mean square residual
VR: virtual reality
