# Supplementary material for: Acceptability Evaluation of the Use of Virtual Reality Games in Smoking-Prevention Education for High School Students: Prospective Observational Study
Source: J Med Internet Res. 2021 Sep 28;23(9):e28037. doi: 10.2196/28037 (PMC8512192; doi:10.2196/28037)
Supplement: Multimedia Appendix 1 [file jmir_v23i9e28037_app1.docx]

**Multimedia Appendix 1.** Theoretical concepts used in the design of the educational games.

| **Motivating concepts** | **Persuasive strategies** | **Examples of the educational games** |
| --- | --- | --- |
|  |  |  |
| **Attention**  Students’ attention is directed to the stimuli, and their interest is maintained over time. | **Competition**  Inclusion of an element of competition in regard to performing the desired behavior. | 1. **Whack-a-mole game**   Students need to stay focused and hit representations of cigarettes.  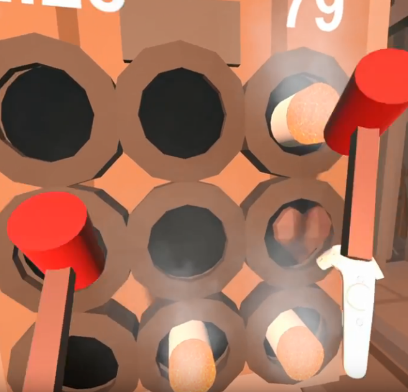   1. **The wire loop game**   Students move an electric pen through a loop without touching the sides of the loop. This requires high focus and concentration on keeping one’s hands steady.  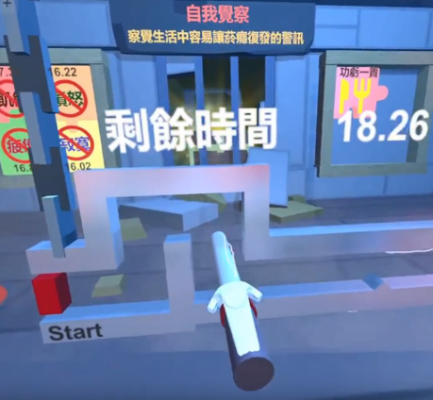 |
| **Relevance**  Students perceive the contents as being related to their real-life experiences. | **Self-awareness**  Inclusion of information that raises awareness of the physical experiences that are associated with risk of smoking. | 1. **The wire loop game**   At the start point, students select a physical experience (eg, tiredness). At the end point, they learn that this physical experience can increase the risk of smoking. Four common physical experiences are available for selection: hunger, anger, tiredness, loneliness.  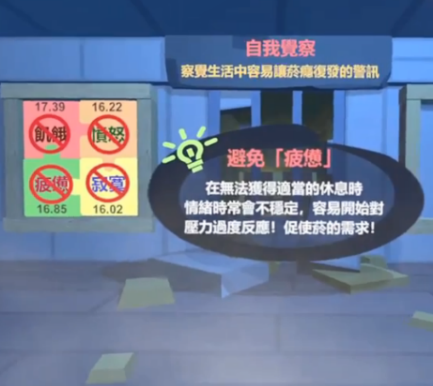   1. **Square Baseball Game**   (2 × 2 Squared Up)  Students throw four baseballs to repel risks.  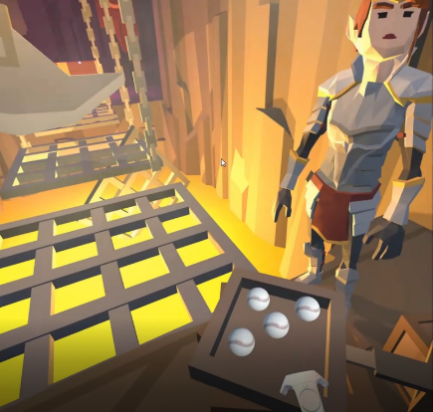 |
| **Confidence**  Confident students attribute their successes in the games to their own ability and efforts. | **Feedback**  Inclusion of information that allows students to track their own achievements in the game, providing details on both past and current performance. | 1. **Taiko drum**   Students are presented with statistics describing their performance in the task. The statistics include three achievement levels (“perfect,” “good,” and “normal”), number of missed points, total number of points scored, and number of consecutive hits.)  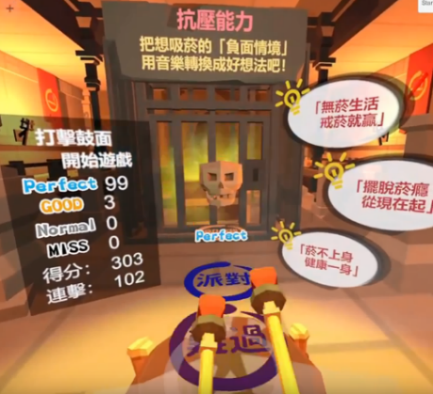 |
|  | **Social comparison**  Inclusion of a means by which students can view others’ performances and compare them with their own. | 1. **Whack-a-mole game**   If students score above average, a congratulatory statement is shown to indicate their achievement.  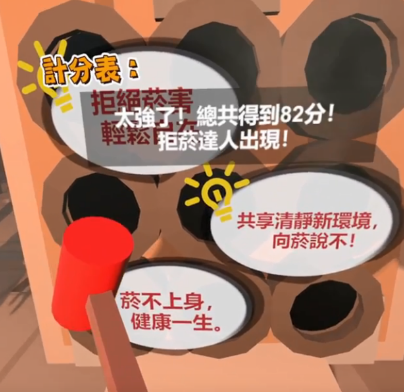 |
| **Satisfaction**  Students perceive the education as inducing internal feelings of satisfaction. | **Reinforcement**  Students are rewarded with upgraded in-game equipment after they complete the challenge tasks. | 1. **Sword: Bravely refuse smoking**   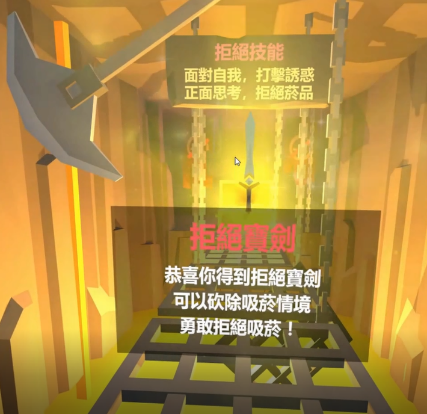  **(2) Armor: Avoid being deceived**  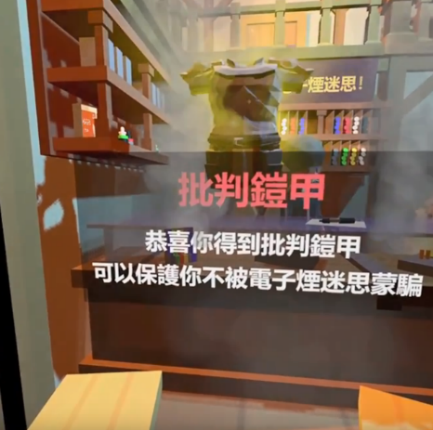 |
| **Knowledge** | **Information**  Provision of accurate information related to e-cigarettes. | **Scenario for breaking the myth of e-cigarettes**  Students enter an e-cigarette store and are approached by a salesman. They answer yes/no questions associated with sales tactics regarding e-cigarettes.  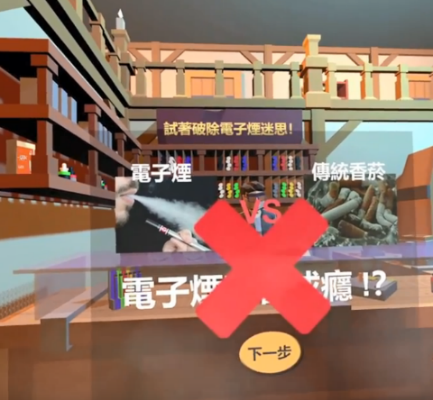 |
